# Heat shock transcription factors in banana: genome-wide characterization and expression profile analysis during development and stress response

**DOI:** 10.1038/srep36864

**Published:** 2016-11-18

**Authors:** Yunxie Wei, Wei Hu, Feiyu Xia, Hongqiu Zeng, Xiaolin Li, Yu Yan, Chaozu He, Haitao Shi

**Affiliations:** 1 Hainan Key Laboratory for Sustainable Utilization of Tropical Bioresources, College of Agriculture, Hainan University, Haikou, 570228, China; 2 Key Laboratory of Biology and Genetic Resources of Tropical Crops, Institute of Tropical Bioscience and Biotechnology, Chinese Academy of Tropical Agricultural Sciences, Xueyuan Road 4, Haikou, Hainan province, 571101, China

## Abstract

Banana (*Musa acuminata*) is one of the most popular fresh fruits. However, the rapid spread of fungal pathogen *Fusarium oxysporum* f. sp. cubense (*Foc*) in tropical areas severely affected banana growth and production. Thus, it is very important to identify candidate genes involved in banana response to abiotic stress and pathogen infection, as well as the molecular mechanism and possible utilization for genetic breeding. Heat stress transcription factors (Hsfs) are widely known for their common involvement in various abiotic stresses and plant-pathogen interaction. However, no *MaHsf* has been identified in banana, as well as its possible role. In this study, genome-wide identification and further analyses of evolution, gene structure and conserved motifs showed closer relationship of them in every subgroup. The comprehensive expression profiles of *MaHsfs* revealed the tissue- and developmental stage-specific or dependent, as well as abiotic and biotic stress-responsive expressions of them. The common regulation of several *MaHsfs* by abiotic and biotic stress indicated the possible roles of them in plant stress responses. Taken together, this study extended our understanding of *MaHsf* gene family and identified some candidate *MaHsfs* with specific expression profiles, which may be used as potential candidates for genetic breeding in banana.

Plant heat shock responses are mediated by heat shock elements (HSEs, nTTCnnGAAnnTTCn), which are widely present in the upstream of the heat shock proteins (HSPs)^1^,^2^,^3^,^4^,^5^. The first specific transcription regulator that is responsible for HSE bindng, was characterized and confirmed as heat stress transcription factor (Hsf) in yeast (*Saccharomyces cerevisiae*)^6^. Thereafter, Hsfs act through the cis-acting element of HSE, thus directly recognize the promoters of HSPs and regulate their transcripts^7^,^8^,^9^.

As evolutionarily conserved transcription factors, Hsfs have some conserved domains. (i) the highly structured N-terminal DNA-binding domain (DBD), that is responsible for binding HSEs in the promoters of several HSPs; (ii) the oligomerization domain (HR-A/B), that is connected to the DBD by a flexible linker; (iii) nuclear export signal (NES) of motif –LFGV- and nuclear localization signal (NLS), (iv) C-terminal activator motic, also known as AHA motif 8,9,10,11,12. According to the flexible linker of variable length (about 15–80 amino acids) and the oligomerization domain (HR-A/B), plant Hsfs can be divided into at least three types, including class A (A1, A2, A3, A4, A5, A6, A7, A8, A9), class B (B1, B2, B3, B4) and class C (C1, C2)^1^,^2^,^9^,^10^,^11^,^12^.

Plant *Hsfs* are important regulators of various plant responses to abiotic and biotic stresses, including heat, cold, salt, drought, osmotic^12^,^13^,^14^,^15^,^16^, as well as bacterial and fungal pathogen infection^17^,^18^,^19^,^20^. After initially identified in yeast^6^, plant *Hsf* gene family has been identified and characterized in more and more plant species, including alfalfa (*Medicago sativa* L.)^21^, *Arabidopsis thaliana*^22^, rice (*Oryza sativa* L.)^23^,^24^, maize (*Zea mays* L.)^25^, *Medicago truncatula, Populus trichocarpa*^26^, wheat (*Triticum aestivum* L.)^27^, soybean (*Glycine max*)^28^,^29^, Chinese cabbage (*Brassica rapa* ssp. *pekinensis*)^10^,^30^, cotton (*Gossypium hirsutum*)^31^, pigeonpea (*Cajanus cajan*), barrel medic (*Medicago truncatula*)^32^, pepper (*Capsicum annuum* L.)^33^,^34^, pear (*Pyrus bretschneideri*)^35^, *Populus euphratica*^36^, strawberry (*Fragaria vesca*)^37^, tea plant (*Camellia sinensis*)^38^, wild Chinese grapevine (*Vitis pseudoreticulata*)^39^, etc.

Banana (*Musa acuminata*) is one of the most popular fresh fruits worldwide, and cultivated in the subtropical and tropical areas^40^,^41^,^42^,^43^. To date, many banana varieties have been screen and cultivated in China. For example, BaXi jiao (*Musa acuminata* L. AAA group cv. Cavendish, BX) is widely cultivated for its high yield, long fingers and long-term storage; Fen jiao (*Musa* ABB Pisang Awak, FJ) is widely cultivated for its good flavor and good resistant to various abiotic stresses^44^,^45^. However, because of the rapid spread of fungal pathogen *Fusarium oxysporum* f. sp. cubense (*Foc*) in tropical areas, banana growth and production are severely affected^46^,^47^,^48^,^49^,^50^,^51^,^52^,^53^,^54^,^55^,^56^,^57^,^58^. Thus, it is very important to identify candidate genes involved in banana response to both high temperature and pathogen infection, as well as the molecular mechanism and possible utilization for genetic breeding. Hsfs are widely known for their common involvement in various abiotic stresses including heat stress and plant-pathogen interaction. However, no *MaHsf* have been identified in banana, as well as their possible roles. In this study, genome-wide identification and expression analysis during development and stress response were performed to extend our understanding and possible utilization of *MaHsfs* in genetic breeding.

## Results

### Genome-wide identification of *Hsfs* in banana

After initial identification using *Musa acuminata v1* Phytozome database v10.3 and Plant Transcription Factor Database (PlantTFDB) v3.0^59^ as well as further confirmation using National Center for Biotechnology Information (NCBI)’s conserved domain database (CDD)^60^ and Pfam database^61^, 43 *MaHsfs* were successfully obtained from banana genome (Supplementary Table S1). The amino acid residues, molecular weight (MW) and theoretical isoelectric point (pI) of 43 MaHsf proteins were largely different, ranging from 96 aa/10.25 kDa (MaHsf12) to 547 aa/60.12 kDa (MaHsf8), 4.48 pI (MaHsf8) to 11.84 pI (MaHsf39) (Supplementary Table S1).

### Phylogenetic analysis of *MaHsfs*

**Figure 1:**
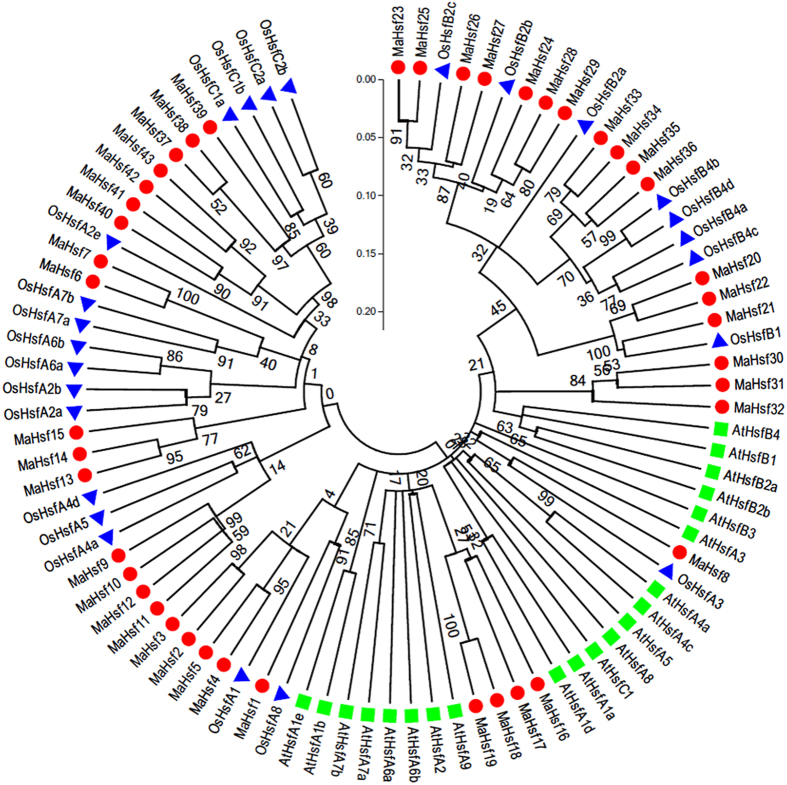
The phylogenetic tree of 43 *MaHsfs, 22 AtHsfs* and *25 OsHsfs* that was constructed using MEGA5.05 software.

To investigate the evolutionary relationship among *Hsfs* from cassava, *Arabidopsis* and rice, an un-rooted Neighbor-Joining tree was created based on the coding sequences of 43 *MaHsfs*, 22 *AtHsfs* and 25 *OsHsfs* (Fig. 1). Generally, *MaHsfs* have closer relationship with *OsHsfs* in comparison to *AtHsfs*, in accordance with the current understanding in their evolutionary history. Evolutionary analysis also identified some orthologous *Hsfs* between cassava and rice, indicating the similar roles of these genes in cassava and *Arabidopsis*.

### Gene structure and conserved motif analysis of *MaHsfs*

**Figure 2:**
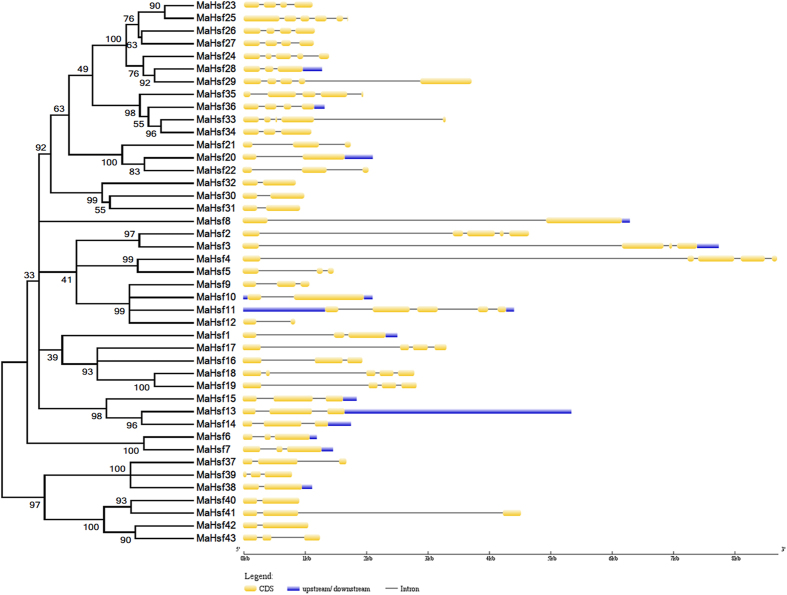
Gene structure analysis of 43 *MaHsfs*. The relationship of gene evolution and structure was analyzed using MEGA5.05 and GSDS v2.0.

**Figure 3:**
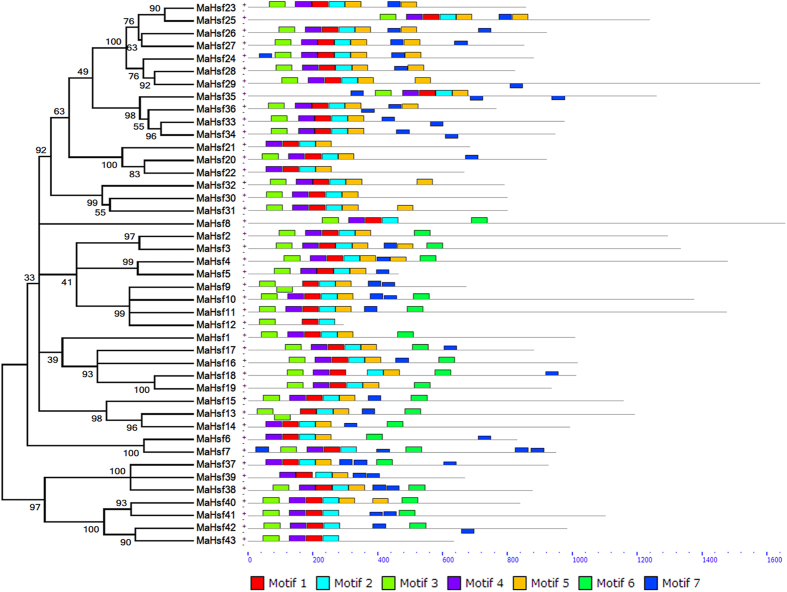
The conserved motif analysis of 43 *MaHsfs*. The relationship of gene evolution and motifs was analyzed using MEGA5.05 and MEME v4.11.0.

To reveal the structural features of *MaHsfs*, intron/exon and upstream (5′ UTR)/downstream (3′ UTR) structures were analyzed using Gene Structure Display Server (GSDS) v2.0^62^. The numbers of intron of *MaHsfs* varied from 1 to 5 (Fig. 2). Most of *MaHsfs* (29 of 43) have no upstream and downstream sequences, only *MaHsf10* and *MaHsf11* have both upstream and downstream sequences, and 12 *MaHsfs* have only downstream sequences (Fig. 2). Moreover, *MaHsfs* in the same subfamilies exhibited similar exon-intron structure, indicating the link between evolutionary relationship and gene structure. To better understand the functional prediction of *MaHsfs*, 7 conserved motifs were identified using Multiple Em for Motif Elicitation (MEME) v4.11.0 (Fig. 3). Similarly, *MaHsfs* in the same subfamilies showed similar motifs, indicating the link between evolutionary relationship and conserved motifs.

### Expression analysis of *MaHsfs* in different banana tissues

**Figure 4:**
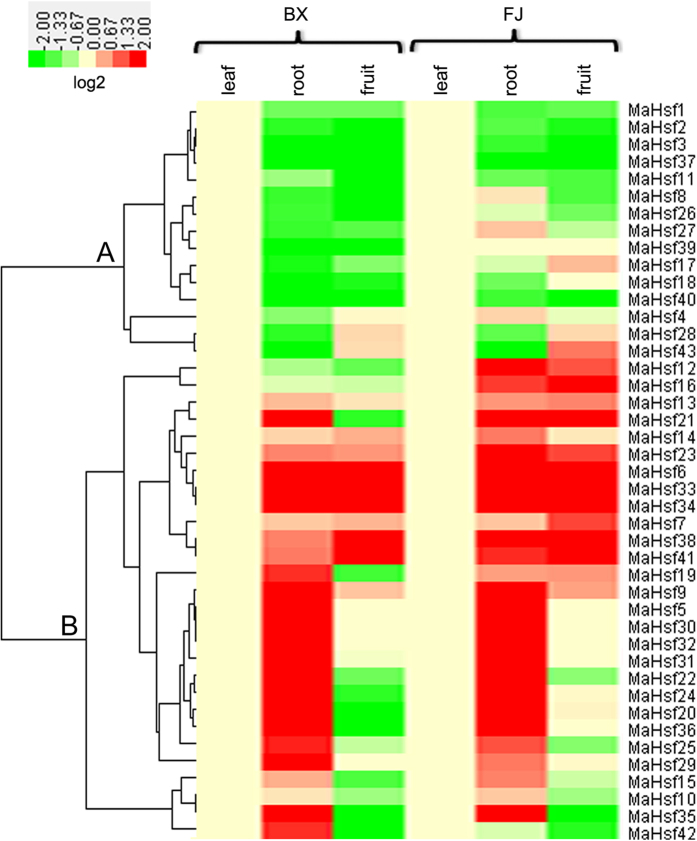
Gene expression heatmap of *MaHsfs* in banana leaves, roots and fruits. The samples of different banana organs were harvested from five-leaf stage leaves, roots and fruits of 80 DAF as Hu *et al*. (2015b,c) described. The heatmap was constructed using CLUSTER software and Java Treeview software.

The expression levels of *MaHsfs* are important clues for their possible roles in banana growth and development, and the transcripts of *MaHsfs* in five-leaf stage leaves, roots and fruits of 80 days after flowering (DAF) were obtained by transcriptomic analysis^44^,^45^. Generally, *MaHsfs* showed similar expression pattern in leaves, roots and fruits of BX and FJ varieties, with litter differences in several genes (14 of 43 genes) (*MaHsf8, 27, 17, 4, 43, 12, 16, 21, 14, 19, 24, 20, 36, 42*) (Fig. 4). The MaHsfs with similar expression pattern can be clearly shown in the cluster analysis (Fig. 4).

For BX varieties, (i) 16 of 43 *MaHsfs* displayed higher transcripts in five-leaf stage leaves, relative lower transcripts in roots and fruits (cluster A). (ii) 19 of 43 *MaHsfs* exhibited relative higher transcripts in roots, and 8 of 43 *MaHsfs* showed lower transcripts in fruits (cluster B).

For FJ varieties, (i) 11 of 43 *MaHsfs* displayed higher transcripts in five-leaf stage leaves, relative lower transcripts in roots or fruits (cluster A). (ii) 22 of 43 *MaHsfs* exhibited relative higher transcripts only in roots, 9 of 43 *MaHsfs* exhibited relative higher transcripts in both roots and fruits, 1 of 43 *MaHsfs* showed lower transcripts in fruits (cluster B).

### Expression analysis of *MaHsfs* in different developmental stages of fruit development and ripening of banana

**Figure 5:**
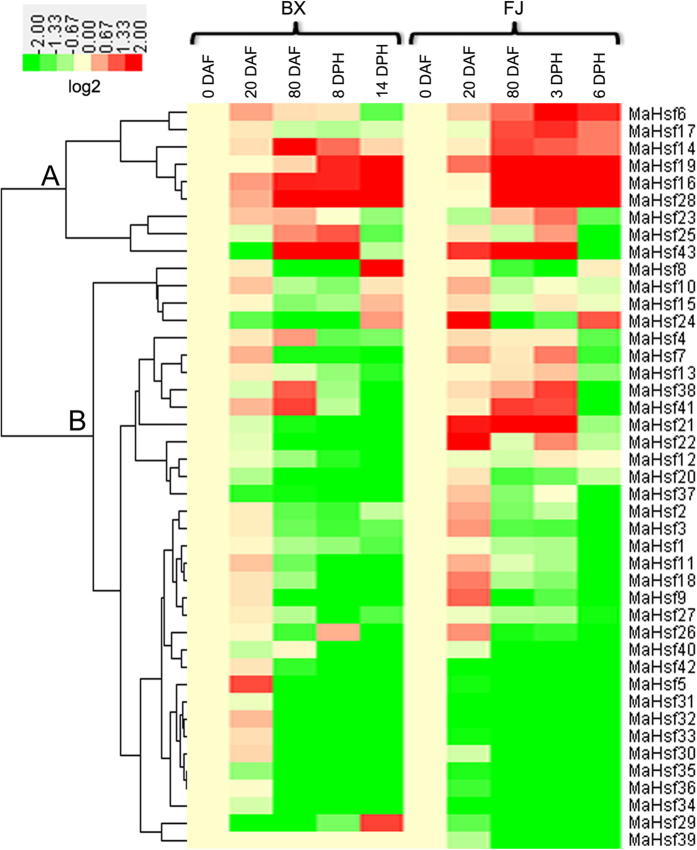
Gene expression heatmap of *MaHsfs* in different developmental stages. The samples of different stages of fruit development and ripening were harvested from fruits of 0, 20, 80 DAF, 8 and 14 days (BX) or 3 and 6 days (FJ) DPH) as Hu *et al*. (2015b,c) described. The heatmap was constructed using CLUSTER software and Java Treeview software.

Besides different banana tissues, the transcripts of *MaHsfs* in different stages of fruit development (0, 20, 80 DAF) and ripening (8 and 14 days (BX) or 3 and 6 days (FJ) postharvest (DPH)) were also analyzed by transcriptomic analysis^44^,^45^. Although some slight differences were exhibited, *MaHsfs* showed similar expression pattern in different stages of fruit development and ripening of BX and FJ varieties, as evidenced by the cluster analysis in the heatmap (Fig. 5). In cluster A, most of *MaHsfs* exhibited relative higher transcript accumulation in fruit development and ripening, and three *MaHsfs (MaHsf23, 25, 43*) showed decreased transcripts in later fruit ripening in both BX and FJ varieties (Fig. 5A). In cluster B, most of *MaHsfs* showed relative lower transcripts in fruit development and ripening in both BX and FJ varieties, while *MaHsf39* showed no significant difference in these stages of BX, 6 *MaHsfs (MaHsf7, 12, 21, 22, 38, 42*) displayed relative higher transcripts in fruit development and early fruit ripening (Fig. 5B).

### Expression analysis of *MaHsfs* in response to cold, salt and osmotic stresses

**Figure 6:**
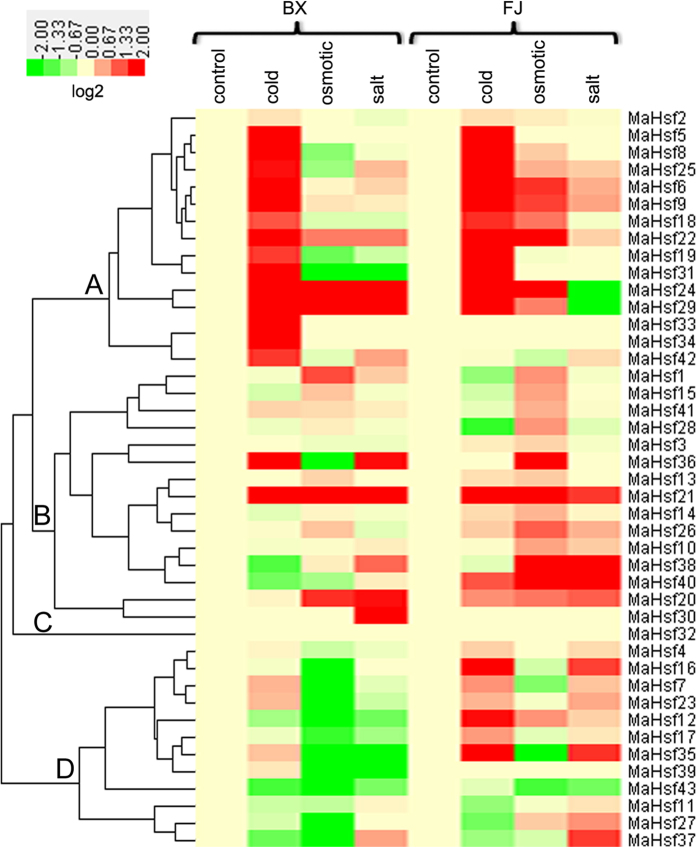
Gene expression heatmap of *MaHsfs* in response to cold, salt and osmotic stresses. Five-leaf stage banana seedlings were treated by 4 °C for 22 h, 300 mM NaCl for 7 d, or 200 mM Mannitol for 7 d as Hu *et al*. (2015b,c) described. The heatmap was constructed using CLUSTER software and Java Treeview software.

To extend our understanding of *MaHsfs* in response to abiotic stress, the expression patterns of these genes in response to cold, salt and osmotic stresses were also revealed by transcriptomic analysis^44^,^45^. Although some similar expression patterns were exhibited (cluster A, B and C), *MaHsfs* showed complex expression patterns in response to abiotic stress in BX and FJ varieties, as evidenced by the cluster analysis in the heatmap (Fig. 6).

**Figure 7:**
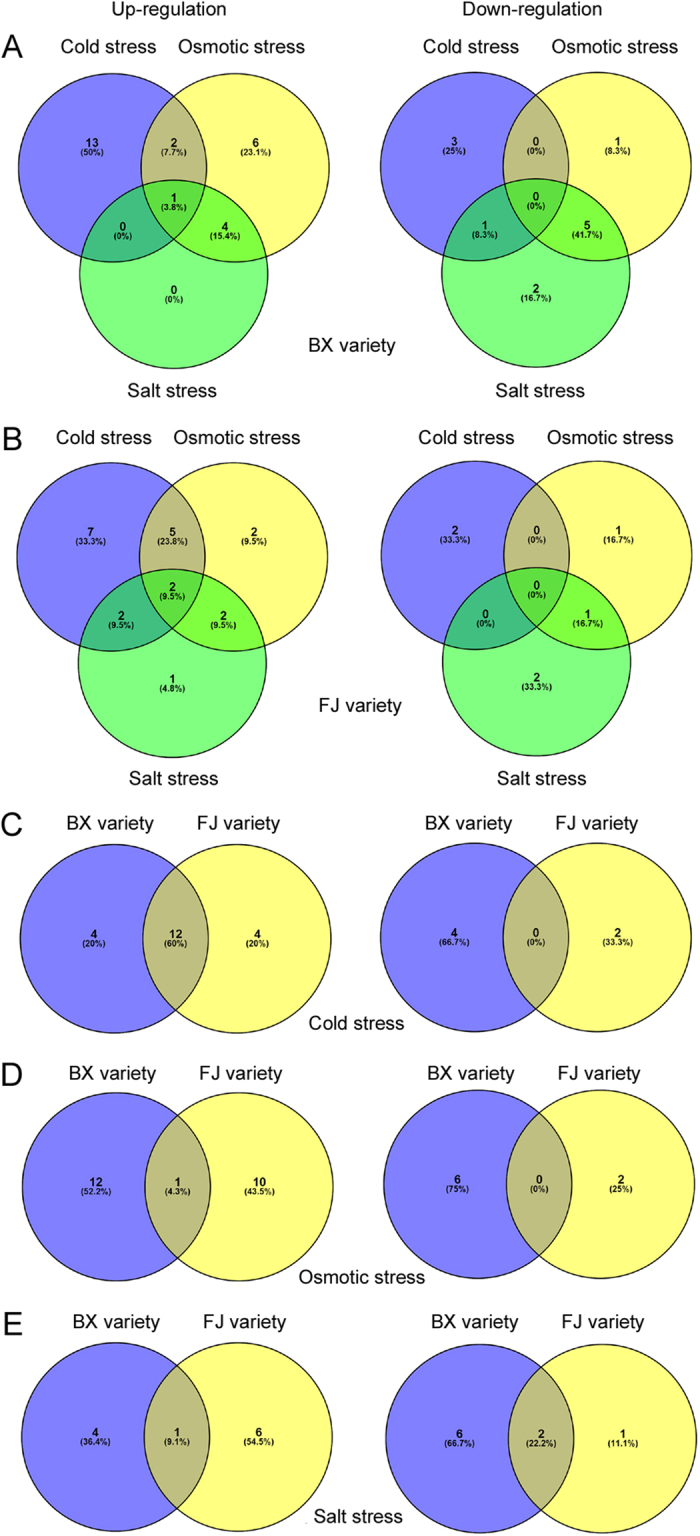
Vene diagram showing the number of overlapping *MaHsfs* that are differentially expressed under cold, salt and osmotic stresses. (**A**,**B**) Vene diagram showing the number of differentially expressed in response to cold, salt and osmotic stresses in BX variety (**A**) and FJ variety (**B**). (**C**–**E**) Vene diagram showing the number of differentially expressed in response to cold stress (**C**), osmotic stress (**D**) and salt stress (**E**) in both BX and FJ varieties.

For BX varieties, 20, 19 and 13 *MaHsfs* were regulated by cold stress (16 up-regulated genes and 4 down-regulated genes), osmotic stress (13 up-regulated genes and 6 down-regulated genes) and salt stress (5 up-regulated genes and 8 down-regulated genes), respectively (Figs 6 and 7A). Among these genes, *MaHsf31* transcript was commonly up-regulated by cold, salt and osmotic stresses, *MaHsf19* and *MaHsf36* transcripts were commonly up-regulated by cold and osmotic stresses, *MaHsf12, 35, 39* and *43* transcripts were commonly up-regulated by salt and osmotic stresses (Figs 6 and 7A). On the contrary, *MaHsf38* transcript was commonly down-regulated by cold and salt stresses, *MaHsf20, 21, 22, 24* and *29* transcripts were commonly down-regulated by salt and osmotic stresses (Figs 6 and 7A).

For FJ varieties, 18, 13 and 10 *MaHsfs* were regulated by cold stress (16 up-regulated genes and 2 down-regulated genes), osmotic stress (11 up-regulated genes and 2 down-regulated genes) and salt stress (7 up-regulated genes and 3 down-regulated genes), respectively (Figs 6 and 7B). Among these genes, *MaHsf21* and *MaHsf40* transcripts were commonly up-regulated by cold, salt and osmotic stresses, *MaHsf6, 9, 18, 22* and *24* transcripts were commonly up-regulated by cold and osmotic stresses, *MaHsf16* and *MaHsf35* transcripts were commonly up-regulated by cold and salt stresses, *MaHsf20* and *MaHsf38* transcripts were commonly up-regulated by salt and osmotic stresses (Figs 6 and 7B). On the contrary, *MaHsf43* transcript was commonly down-regulated by salt and osmotic stresses (Figs 6 and 7B).

Moreover, we also found the transcripts of some *MaHsfs* were regulated by the same stress in both BX and FJ varieties (Fig. 7C–E). For cold stress, 12 *MaHsfs (MaHsf5, 6, 8, 9, 18, 19, 21, 22, 24, 25, 29, 31*) transcripts were up-regulated in both BX and FJ varieties (Fig. 7C). For osmotic stress, *MaHsf36* transcript was up-regulated in both BX and FJ varieties (Fig. 7D). For salt stress, *MaHsf35* transcript was up-regulated, *MaHsf24* and *MaHsf29* transcripts were down-regulated in both BX and FJ varieties (Fig. 7E).

### Identification of several *MaHsfs* responsive to *Foc1* and *Foc 4* inoculation

**Figure 8:**
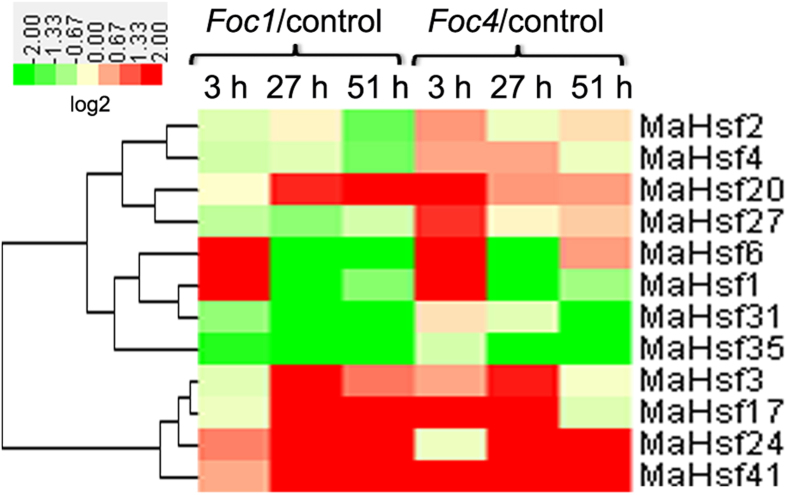
Gene expression heatmap of *MaHsfs* in response to *Foc1* and *Foc 4* inoculation. The samples were harvested from banana roots that were inoculated by control, *Foc1* or *Foc4* for 3 h, 27 h and 51 h. The heatmap was constructed using CLUSTER software and Java Treeview software.

The rapid spread of fungal pathogen *Foc* in tropical areas severely affects banana growth and production^46^,^47^,^48^,^49^,^50^,^51^,^52^,^53^,^54^,^55^,^56^,^57^,^58^. To investigate the possible involvement and utilization of *MaHsfs* in plant-pathogen interaction, the transcriptomic analysis of banana roots in response to control, *Foc1* or *Foc4* were also obtained^50^. Totally, 12 of 43 *MaHsfs* were significantly regulated by *Foc* infection (Fig. 8). Among these genes, 5 *MaHsfs (MaHsf3, 17, 20, 24, 41*) were commonly up-regulated by *Foc 1* and *Foc 4*, 2 *MaHsfs (MaHsf31, 35*) were commonly down-regulated by *Foc 1* and *Foc 4* (Fig. 8). *MaHsf1* and *MaHsf6* were first up-regulated and later down-regulated by *Foc 1* and *Foc 4*, whereas *MaHsf2* and *MaHsf4* were down-regulated by *Foc 1*, but were up-regulated by *Foc 4* (Fig. 8).

## Discussion

As one of the most popular fresh fruits worldwide, banana are severally destroyed by various abiotic stress (cold, salt, drought, etc) and biotic stress (especially the fungal pathogen *Foc* infection) during growth and developmental stages^46^,^47^,^48^,^49^,^50^,^51^,^52^,^53^,^54^,^55^,^56^,^57^,^58^. To solve these questions, the farmers and researchers have increased the planting area and improved cultivated technique, however, the effect is very limited. Because no strong stress-resistant banana variety can be used, it is essential to construct new stress-resistant variety through genetic and molecular breeding^46^,^47^,^48^,^49^,^50^,^51^,^52^,^53^,^54^,^55^. Considering the common involvement of *Hsfs* in plant stress responses, *MaHsfs* were chosen for analyzed as candidate genes for further utilization in genetic breeding.

Generally, 43 *MaHsfs* were genome-wide identified and the phylogenetic evolution of these genes was also revealed. Based on the data of RNA-seq^44^,^45^,^50^, the comprehensive expression profiles of 43 *MaHsfs* were revealed. To our knowledge, this is the first study extending our understanding of *MaHsf* gene family. Generally, the expression profiles can be divided to two sections. One is the expression of 43 *MaHsfs* at developmental stages, or different tissues, this is the basic information of this gene family. We found that the transcripts of some *MaHsfs* are tissue- and developmental stage-specific or dependent, indicating the possible roles of them in specific growth or developmental stages, such as fruit ripening. The other one gene expression in response to various abiotic and biotic stresses, which intends to identify several candidate genes commonly regulated by various stresses for stress-related genetic breeding.

In this study, multiple of abiotic and biotic stress-responsive *MaHsfs* were also identified, in accordance with previous studies of *Hsf* gene family in other plant species^1^,^2^,^3^,^4^,^5^. Based on the clues from transcript pattern, the *in vivo* roles of several plant *Hsfs* have been revealed. *AtHsfA1s, AtHsfA2* and *AtHsfA6a* confers heat, salt and dehydration stress resistance in *Arabidopsis*^3^,^5^,^7^,^8^. *OsHSFA2dI* is essential for heat stress resistance in rice^1^, respectively. *AtHsfA6a* confers salt and dehydration stress resistance in *Arabidopsis*^3^. A seed preferential *TaHsf* confers abiotic stress tolerance in *Arabidopsis*^27^, and overexpression of *GmHsf-24* increased drought and heat stress resistance in *Arabidopsis*^29^. These results provide solid evidence of the protective roles of plant *Hsfs* in abiotic stress responses. In this study, 12 *MaHsfs* (12, 19, 20, 21, 22, 24, 29, 31, 35, 36, 39, 43) in BX variety and 10 *MaHsfs* (6, 9, 16, 18, 20, 22, 24, 35, 38, 43) in FJ variety were commonly regulated by at least two abiotic stresses (fold change >2), indicating their possible roles in abiotic stress. Thus, these MaHsfs can be further chosen for functional analysis.

Additionally, plant *Hsfs* are also involved in plant-pathogen interactions^17^,^18^,^19^,^20^. The activation of OsHsf23 is important for cell death in rice inoculated with rice blast fungus^20^. *AtHsfA1b, AtHsfB1* and *AtHsfB2b* are involved in plant pathogen resistance and defense gene expression^17^,^19^. Thus, the identification of 12 *Foc-*responsive *MaHsfs* (1, 2, 3, 4, 6, 17, 20, 24, 27, 31, 35, 41) may be further used as potential candidates in disease genetic breeding in banana. Notably, *MaHsf31* transcript was commonly up-regulated by cold, salt and osmotic stresses in BX, and was down-regulated by *Foc 1* and *Foc 4* infection; *MaHsf20* and *MaHsf24* transcripts were commonly regulated by salt stress, osmotic stress and *Foc* infection. There are also other *MaHsfs* that are regulated by both abiotic and biotic stresses, such as *MaHsf35*. The common regulation of these *MaHsfs* by abiotic and biotic stress indicated the possible roles of them in plant stress responses, and these *MaHsfs* may be potential candidates for further functional analysis and genetic breeding in banana. We highlight the possible common involvement of four *MaHsfs (MaHsf20, MaHsf24, MaHsf31* and *MaHsf35*) in both abiotic and biotic stresses. It is just the beginning, further functional analysis will reveal their *in vivo* roles as well as underlying mechanism.

Taken together, this study is the first study showing *MaHsf* gene family as well as their specific expression profiles, which may be used as potential candidates for genetic breeding in banana.

## Methods

### Plant materials and growth conditions

Banana varieties of BX and FJ were used in this study. The five leaf stage seedlings were from Banana Tissue Culture Center (Danzhou city, Hainan province, Institute of Banana and Plantains, Chinese Academy of Tropical Agricultural Sciences). Thereafter, the seedlings were cultivated in soil in the greenhouse, which was controlled at 28 °C, with 12 h light/12 h dark cycles and 120–150 μmol quanta m^−2^ s^−1^ irradiance.

### Genome-wide identification of *MaHsfs*

The candidate *MaHsfs* were first searched in *Musa acuminata v1* (Banana) Phytozome database v10.3 (https://phytozome.jgi.doe.gov/pz/portal.html#!info?alias=Org_Macuminata) and PlantTFDB v3.0 (http://planttfdb.cbi.pku.edu.cn/index.php)^59^. Thereafter, the candidate *MaHsfs* were further checked and confirmed using CDD (http://www.ncbi.nlm.nih.gov/cdd)^60^ and Pfam database (http://pfam.xfam.org)^61^. Then the detailed information of *MaHsfs* including sequences, the locus name, chromosome location, gene and amino acid length were downloaded from *Musa acuminata v1* (Banana) Phytozome database v10.3, the MW and pI of *MaHsfs* were analyzed using ProtParam software (http://web.expasy.org/protparam).

### Phylogenetic analysis of *MaHsfs*

Besides the coding sequences of 43 *MaHsfs*, the coding sequences of 22 *AtHsfs* and 25 *OsHsfs* were obtained from hytozome database v10.3 (https://phytozome.jgi.doe.gov/pz/portal.html). The phylogenetic tree of these *Hsfs* was constructed using Clustalx 1.83 software and MEGA 5.05 software by the neighbor-joining method^63^,^64^.

### Gene structure and conserved motif analysis of *MaHsfs*

Gene structure analysis of 43 *MaHsfs* was performed using GSDS v2.0 (http://gsds.cbi.pku.edu.cn/index.php) by uploading the coding sequences and genomic sequences of these genes^62^. The conserved motifs of 43 *MaHsfs* were analyzed using the MEME v4.11.0 (http://meme-suite.org/tools/meme) by uploading the coding sequences according the instructions.

### Expression profile analysis of *MaHsfs*

The transcriptomic data of two banana varieties (BX and FJ) in different organs, different developmental stages and in response to abiotic stress has been described in Hu *et al*.^44^,^45^. Briefly, the different organs include five-leaf stage leaves, roots and fruits of 80 DAF, different developmental stages include fruits of 0, 20, 80 DAF, 8 and 14 (BX) or 3 and 6 (FJ) DPH, abiotic stress treatment include 4 °C treatment for 22 h, 300 mM NaCl treatment for 7 d and 200 mM Mannitol treatment for 7 d.

The transcriptomic data of roots in response to pathogen fungal pathogen of *Foc1* and *Foc 4* has been described previously^50^. For the gene expression assay, sterile tissue cultivated banana roots were inoculated by control, *Foc1* or *Foc4* for 3 h, 27 h and 51 h.

### Hierarchical cluster and gene expression heatmap analysis

The hierarchical cluster analysis of gene expression was performed using CLUSTER software (http://bonsai.hgc.jp/~mdehoon/software/cluster/software.htm)^63^, and the heatmap was then constructed using Java Treeview software (http://jtreeview.sourceforge.net)^65^.

## Additional Information

**How to cite this article**: Wei, Y. *et al*. Heat shock transcription factors in banana: genome-wide characterization and expression profile analysis during development and stress response. *Sci. Rep.* **6**, 36864; doi: 10.1038/srep36864 (2016).

**Publisher’s note**: Springer Nature remains neutral with regard to jurisdictional claims in published maps and institutional affiliations.

## Supplementary Material

Supplementary Information

## References

[b1] Cheng Q. . An alternatively spliced heat shock transcription factor, OsHSFA2dI, functions in the heat stress-induced unfolded protein response in rice. Plant Biol (Stuttg). 17, 419–429 (2015). 25255693 10.1111/plb.12267

[b2] Giesguth M., Sahm A., Simon S. & Dietz K. J. Redox-dependent translocation of the heat shock transcription factor AtHSFA8 from the cytosol to the nucleus in *Arabidopsis thaliana*. FEBS Lett. 589, 718–725 (2015). 25666709 10.1016/j.febslet.2015.01.039

[b3] Hwang S. M. . Functional characterization of *Arabidopsis* HsfA6a as a heat-shock transcription factor under high salinity and dehydration conditions. Plant Cell Environ. 37, 1202–1222 (2014). 24313737 10.1111/pce.12228

[b4] Kotak S., Port M., Ganguli A., Bicker F. & von Koskull-Döring P. Characterization of C-terminal domains of *Arabidopsis* heat stress transcription factors (Hsfs) and identification of a new signature combination of plant class A Hsfs with AHA and NES motifs essential for activator function and intracellular localization. Plant J. 39, 98–112 (2004). 15200645 10.1111/j.1365-313X.2004.02111.x

[b5] Nishizawa A. . *Arabidopsis* heat shock transcription factor A2 as a key regulator in response to several types of environmental stress. Plant J. 48, 535–547 (2006). 17059409 10.1111/j.1365-313X.2006.02889.x

[b6] Sorger P. K. & Pelham H. R. B. Yeast heat-shock factor is an essential DNA -binding protein that exhibits temperature-dependent phosphorylation. Cell 54, 855–864 (1988). 3044613 10.1016/s0092-8674(88)91219-6

[b7] Charng Y. Y. . A heat-inducible transcription factor, HsfA2, is required for extension of acquired thermotolerance in *Arabidopsis*. Plant Physiol. 143, 251–262 (2007). 17085506 10.1104/pp.106.091322 PMC1761974

[b8] Nishizawa-Yokoi A. . HsfA1d and HsfA1e involved in the transcriptional regulation of HsfA2 function as key regulators for the Hsf signaling network in response to environmental stress. Plant Cell Physiol. 52, 933–945 (2011). 21471117 10.1093/pcp/pcr045

[b9] Singh A. . OsHsfA2c and OsHsfB4b are involved in the transcriptional regulation of cytoplasmic OsClpB (Hsp100) gene in rice (*Oryza sativa* L.). Cell Stress Chaperones 17, 243–254 (2012). 22147560 10.1007/s12192-011-0303-5 PMC3273560

[b10] Huang X. Y. . Genome-wide identification, classification, and analysis of heat shock transcription factor family in Chinese cabbage (*Brassica rapa* pekinensis). Genet. Mol. Res. 14, 2189–2204 (2015). 25867366 10.4238/2015.March.27.5

[b11] Shim D. . Orthologs of the class A4 heat shock transcription factor HsfA4a confer cadmium tolerance in wheat and rice. Plant Cell 21, 4031–4043 (2009). 20028842 10.1105/tpc.109.066902 PMC2814514

[b12] Yabuta Y. Functions of heat shock transcription factors involved in response to photooxidative stresses in *Arabidopsis*. Biosci. Biotechnol. Biochem. 20, 1–10 (2016). 10.1080/09168451.2016.1176515 27095030

[b13] Almoguera C., Personat J. M., Prieto-Dapena P. & Jordano J. Heat shock transcription factors involved in seed desiccation tolerance and longevity retard vegetative senescence in transgenic tobacco. Planta 242, 461–475 (2015). 26021607 10.1007/s00425-015-2336-y

[b14] Stief A., Brzezinka K., Lämke J. & Bäurle I. Epigenetic responses to heat stress at different time scales and the involvement of small RNAs. Plant Signal. Behav. 9, e970430 (2014). 25482804 10.4161/15592316.2014.970430 PMC4622961

[b15] Wang C., Zhang Q. & Shou H. X. Identification and expression analysis of OsHsfs in rice. J. Zhejiang Univ. Sci. B. 10, 291–300 (2009). 19353748 10.1631/jzus.B0820190 PMC2666206

[b16] Yamada K. & Nishimura M. Cytosolic heat shock protein 90 regulates heat shock transcription factor in *Arabidopsis thaliana*. Plant Signal Behav. 3, 660–662 (2008). 19704818 10.4161/psb.3.9.5775 PMC2634549

[b17] Bechtold U. . *Arabidopsis* HEAT SHOCK TRANSCRIPTION FACTORA1b overexpression enhances water productivity, resistance to drought, and infection. J Exp. Bot. 64, 3467–3481 (2013). 23828547 10.1093/jxb/ert185 PMC3733161

[b18] Daurelio L. D. . Characterization of Citrus sinensis transcription factors closely associated with the non-host response to *Xanthomonas campestris* pv. vesicatoria. J. Plant Physiol. 170, 934–942 (2013). 23453188 10.1016/j.jplph.2013.01.011

[b19] Kumar M. . Heat shock factors HsfB1 and HsfB2b are involved in the regulation of Pdf1.2 expression and pathogen resistance in *Arabidopsis*. Mol. Plant 2, 152–165 (2009). 19529832 10.1093/mp/ssn095 PMC2639743

[b20] Tanabe S. . The elicitor-responsive gene for a GRAS family protein, CIGR2, suppresses cell death in rice inoculated with rice blast fungus via activation of a heat shock transcription factor, OsHsf23. Biosci. Biotechnol. Biochem. 80, 145–151 (2015). 26287768 10.1080/09168451.2015.1075866

[b21] Friedberg J. N., Bowley S. R., McKersie B. D., Gurley W. B. & Czarnecka-Verner E. Isolation and characterization of class A4 heat shock transcription factor from alfalfa. Plant Sci. 171, 332–344 (2006). 22980202 10.1016/j.plantsci.2006.04.007

[b22] Guo J. . Genome-wide analysis of heat shock transcription factor families in rice and *Arabidopsis*. J. Genet. Genomics 35, 105–118 (2008). 18407058 10.1016/S1673-8527(08)60016-8

[b23] Chauhan H., Khurana N., Agarwal P. & Khurana P. Heat shock factors in rice (*Oryza sativa* L.): genome-wide expression analysis during reproductive development and abiotic stress. Mol. Genet. Genomics 286, 171–187 (2011). 21792744 10.1007/s00438-011-0638-8

[b24] Jin G. H., Gho H. J. & Jung K. H. A systematic view of rice heat shock transcription factor family using phylogenomic analysis. J. Plant Physiol. 170, 321–329 (2013). 23122336 10.1016/j.jplph.2012.09.008

[b25] Lin Y. X. . Genome-wide identification, classification and analysis of heat shock transcription factor family in maize. BMC Genomics 12, 76 (2011). 21272351 10.1186/1471-2164-12-76 PMC3039612

[b26] Wang F. . Genome-wide analysis of the heat shock transcription factors in *Populus trichocarpa* and *Medicago truncatula*. Mol Biol. Rep. 39, 1877–1886 (2012). 21625849 10.1007/s11033-011-0933-9

[b27] Chauhan H., Khurana N., Agarwal P., Khurana J. P. & Khurana P. A seed preferential heat shock transcription factor from wheat provides abiotic stress tolerance and yield enhancement in transgenic *Arabidopsis* under heat stress environment. PLoS One 8, e79577 (2013). 24265778 10.1371/journal.pone.0079577 PMC3827158

[b28] Chung E., Kim K. M. & Lee J. H. Genome-wide analysis and molecular characterization of heat shock transcription factor family in *Glycine max*. J. Genet. Genomics 40, 127–135 (2013). 23522385 10.1016/j.jgg.2012.12.002

[b29] Li P. S. . Genome-wide analysis of the Hsf family in soybean and functional identification of GmHsf-34 involvement in drought and heat stresses. BMC Genomics 15, 1009 (2014). 25416131 10.1186/1471-2164-15-1009 PMC4253008

[b30] Song X. . Genome-wide identification, classification and expression analysis of the heat shock transcription factor family in Chinese cabbage. Mol. Genet. Genomics 289, 541–551 (2014). 24609322 10.1007/s00438-014-0833-5

[b31] Wang J., Sun N., Deng T., Zhang L. & Zuo K. Genome-wide cloning, identification, classification and functional analysis of cotton heat shock transcription factors in cotton (*Gossypium hirsutum*). BMC Genomics 15, 961 (2014). 25378022 10.1186/1471-2164-15-961 PMC4233062

[b32] Lin Y. . Genome duplication and gene loss affect the evolution of heat shock transcription factor genes in legumes. PLoS One 9, e102825 (2014). 25047803 10.1371/journal.pone.0102825 PMC4105503

[b33] Guo M. . Cloning and expression analysis of heat-shock transcription factor gene CaHsfA2 from pepper (*Capsicum annuum* L.). Genet. Mol. Res. 13, 1865–1875 (2014). 24668674 10.4238/2014.March.17.14

[b34] Guo M. . Genome-wide analysis, expression profile of heat shock factor gene family (CaHsfs) and characterisation of CaHsfA2 in pepper (*Capsicum annuum* L.). BMC Plant Biol. 15, 151 (2015). 26088319 10.1186/s12870-015-0512-7 PMC4472255

[b35] Qiao X. . Genome-wide identification and comparative analysis of the heat shock transcription factor family in Chinese white pear (*Pyrus bretschneideri*) and five other Rosaceae species. BMC Plant Biol. 15, 12 (2015). 25604453 10.1186/s12870-014-0401-5 PMC4310194

[b36] Shen Z. . *Populus euphratica* HSF binds the promoter of WRKY1 to enhance salt tolerance. Plant Sci. 235, 89–100 (2015). 25900569 10.1016/j.plantsci.2015.03.006

[b37] Hu Y. . Identification, isolation, and expression analysis of heat shock transcription factors in the diploid woodland strawberry *Fragaria vesca*. Front Plant Sci. 6, 736 (2015). 26442049 10.3389/fpls.2015.00736 PMC4569975

[b38] Liu Z. W. . Identification, classification, and expression profiles of heat shock transcription factors in tea plant (*Camellia sinensis*) under temperature stress. Gene 576, 52–59 (2016). 26431998 10.1016/j.gene.2015.09.076

[b39] Hu Y. . Identification and expression analysis of heat shock transcription factors in the wild Chinese grapevine (*Vitis pseudoreticulata*). Plant Physiol. Biochem. 99, 1–10 (2016). 26689772 10.1016/j.plaphy.2015.11.020

[b40] Liu J. . Role for the banana AGAMOUS-like gene MaMADS7 in regulation of fruit ripening and quality. Physiol Plant. 155, 217–231 (2015). 25980771 10.1111/ppl.12348

[b41] Liu J. . Banana Ovate family protein MaOFP1 and MADS-box protein MuMADS1 antagonistically regulated banana fruit ripening. PLoS One 10, e0123870 (2015). 25886169 10.1371/journal.pone.0123870 PMC4401719

[b42] Xu Y. . A banana aquaporin gene, MaPIP1;1, is involved in tolerance to drought and salt stresses. BMC Plant Biol. 14, 59 (2014). 24606771 10.1186/1471-2229-14-59 PMC4015420

[b43] Zhang L. . The MaASR gene as a crucial component in multiple drought stress response pathways in *Arabidopsis*. Funct. Integr. Genomics 15, 247–260 (2015). 25414087 10.1007/s10142-014-0415-y

[b44] Hu W. . Genome-wide identification and expression analyses of aquaporin gene family during development and abiotic stress in banana. Int. J. Mol. Sci. 16, 19728–19751 (2015). 26307965 10.3390/ijms160819728 PMC4581322

[b45] Hu W. . The auxin response factor gene family in banana: genome-wide identification and expression analyses during development, ripening, and abiotic stress. Front Plant Sci. 6, 742 (2015). 26442055 10.3389/fpls.2015.00742 PMC4569978

[b46] Bai T. T. . Transcriptome and expression profile analysis of highly resistant and susceptible banana roots challenged with *Fusarium oxysporum* f. sp. cubense tropical race 4. PLoS One 8, e73945 (2013). 24086302 10.1371/journal.pone.0073945 PMC3781162

[b47] Deng G. M. . roteomic analysis of conidia germination in *Fusarium oxysporum* f. sp. cubense tropical race 4 reveals new targets in ergosterol biosynthesis pathway for controlling Fusarium wilt of banana. Appl. Microbiol. Biotechnol. 99, 7189–7207 (2015). 26129952 10.1007/s00253-015-6768-x

[b48] Guo L. . Genome and transcriptome analysis of the fungal pathogen *Fusarium oxysporum* f. sp. cubense causing banana vascular wilt disease. PLoS One 9, e95543 (2014). 24743270 10.1371/journal.pone.0095543 PMC3990668

[b49] Li C. Y. . Transcriptome profiling of resistant and susceptible Cavendish banana roots following inoculation with *Fusarium oxysporum* f. sp. cubense tropical race 4. BMC Genomics 13, 374 (2012). 22863187 10.1186/1471-2164-13-374 PMC3473311

[b50] Li C. . Analysis of banana transcriptome and global gene expression profiles in banana roots in response to infection by race 1 and tropical race 4 of *Fusarium oxysporum* f. sp. cubense. BMC Genomics 14, 851 (2013). 24304681 10.1186/1471-2164-14-851 PMC4046742

[b51] Li X., Bai T., Li Y., Ruan X. & Li H. Proteomic analysis of *Fusarium oxysporum* f. sp. cubense tropical race 4-inoculated response to Fusarium wilts in the banana root cells. Proteome Sci. 11, 41 (2013). 24070062 10.1186/1477-5956-11-41 PMC3850410

[b52] Ploetz R. C. Fusarium wilt of banana is caused by several pathogens referred to as *Fusarium oxysporum* f. sp. cubense. Phytopathology 96, 653–656 (2006). 18943184 10.1094/PHYTO-96-0653

[b53] Ploetz R. C. Fusarium Wilt of Banana. Phytopathology 105, 1512–1521 (2015). 26057187 10.1094/PHYTO-04-15-0101-RVW

[b54] Ploetz R. C., Kema G. H. & Ma L. J. Impact of diseases on export and smallholder production of banana. Annu. Rev. Phytopathol. 53, 269–288 (2015). 26002290 10.1146/annurev-phyto-080614-120305

[b55] Silva P. R. . Development of a thematic collection of Musa spp accessions using SCAR markers for preventive breeding against *Fusarium oxysporum* f. sp cubense tropical race 4. Genet. Mol. Res. 15, doi: 10.4238/gmr.15017765. (2016). 26985964

[b56] Tan D. . Identification of an endophytic antifungal bacterial strain isolated from the Rubber tree and its application in the biological control of banana Fusarium Wilt. PLoS One 10, e0131974 (2015). 26133557 10.1371/journal.pone.0131974 PMC4489675

[b57] Wang Z. . De novo characterization of the banana root transcriptome and analysis of gene expression under *Fusarium oxysporum* f. sp. Cubense tropical race 4 infection. BMC Genomics 13, 650 (2012). 23170772 10.1186/1471-2164-13-650 PMC3534498

[b58] Wu Y. . Systemic acquired resistance in Cavendish banana induced by infection with an incompatible strain of *Fusarium oxysporum* f. sp. cubense. J. Plant Physiol. 170, 1039–1046 (2013). 23702248 10.1016/j.jplph.2013.02.011

[b59] Jin J. P., Zhang H., Kong L., Gao G. & Luo J. C. PlantTFDB 3.0: a portal for the functional and evolutionary study of plant transcription factors. Nucleic Acids Res. 42, D1182–D1187 (2014). 24174544 10.1093/nar/gkt1016 PMC3965000

[b60] Marchler-Bauer A. . CDD: NCBI’s conserved domain database. Nucleic Acids Res. 43, D222–D226 (2015). 25414356 10.1093/nar/gku1221 PMC4383992

[b61] Finn R. D. . The Pfam protein families database: towards a more sustainable future. Nucleic Acids Res. 44, D279–D285 (2016). 26673716 10.1093/nar/gkv1344 PMC4702930

[b62] Hu B. . GSDS 2.0: an upgraded gene feature visualization server. Bioinformatics 31, 1296–1297 (2015). 25504850 10.1093/bioinformatics/btu817 PMC4393523

[b63] Larkin M. A. . Clustal W and Clustal X version 2.0. Bioinformatics 23, 2947–2948 (2007). 17846036 10.1093/bioinformatics/btm404

[b64] Tamura K. . MEGA5: molecular evolutionary genetics analysis using maximum likelihood, evolutionary distance, and maximum parsimony methods. Mol. Biol. Evol. 28, 2731–2739 (2011). 21546353 10.1093/molbev/msr121 PMC3203626

[b65] Saldanha A. J. Java Treeview-extensible visualization of microarray data. Bioinformatics 20, 3246–3248 (2004). 15180930 10.1093/bioinformatics/bth349

