# Supplementary material for: Heat shock transcription factors in banana: genome-wide characterization and expression profile analysis during development and stress response
Source: Sci Rep. 2016 Nov 18;6:36864. doi: 10.1038/srep36864 (PMC5114564; doi:10.1038/srep36864)
Supplement: Supplementary Information [file srep36864-s1.pdf]

Heat shock transcription factors in banana: genome-wide characterization and expression profile analysis during development and stress response

Yunxie Wei[1,#], Wei Hu[2,#], Feiyu Xia[1], Hongqiu Zeng[1], Xiaolin Li[1], Yu Yan[1], Chaozu He[1,*], Haitao Shi[1,*]

[1] Hainan Key Laboratory for Sustainable Utilization of Tropical Bioresources, College of Agriculture, Hainan University, Haikou, 570228, China

[2] Key Laboratory of Biology and Genetic Resources of Tropical Crops, Institute of Tropical Bioscience and Biotechnology, Chinese Academy of Tropical Agricultural Sciences, Xueyuan Road 4, Haikou, Hainan province, 571101, China

[#] These authors contributed equally to this work.

[*]Correspondence and requests for materials should be addressed to H. S. (email: haitaoshi@hainu.edu.cn) and C. H. (czhe@hainu.edu.cn)

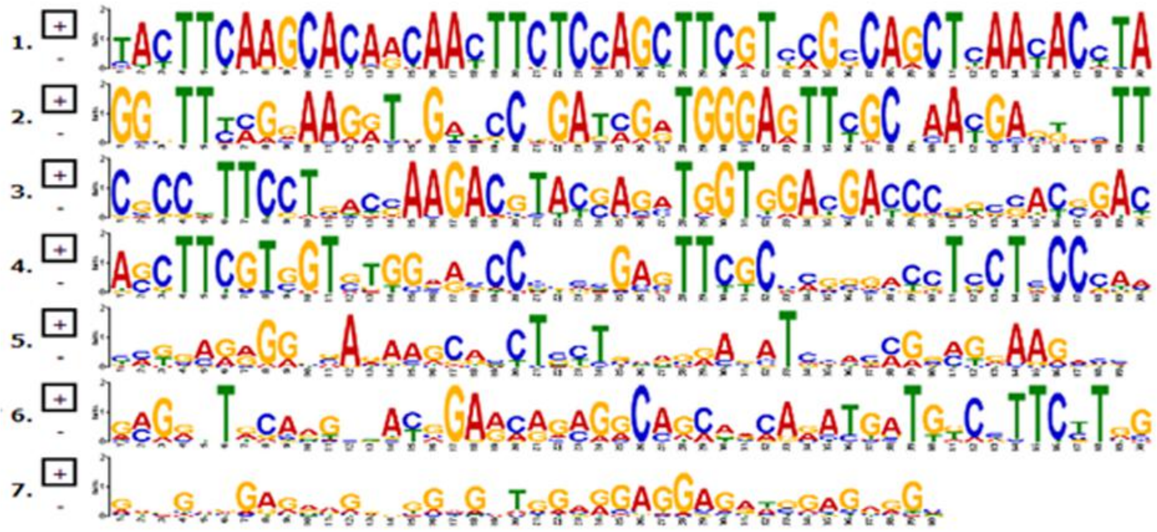

Supplementary Figure S1. The conserved motifs identified in *MaHsfs* by MEME analysis.

Supplementary Table S1. The comprehensive identification of 43 *MaHsfs*.

Gene	Locus Name	Location	CDS (bp)	Amino acids	MW (kDa)	pI
MaHsf1	GSMUA_Achr2G21640_001	chr2:21031681..21034199	999	332	37.44	5.40
MaHsf2	GSMUA_Achr3G27600_001	chr3:27132751..27137417	1281	426	47.51	4.64
MaHsf3	GSMUA_Achr4G04500_001	chr4:3559855..3567615	1320	439	49.07	4.76
MaHsf4	GSMUA_Achr4G17040_001	chr4:15760889..15769596	1464	487	54.62	5.06
MaHsf5	GSMUA_Achr3G06020_001	chr3:4057382..4058867	459	152	16.69	10.51
MaHsf6	GSMUA_Achr2G16660_001	chr2:17459604..17460808	822	273	31.59	5.55
MaHsf7	GSMUA_Achr6G00960_001	chr6:657409..658878	942	313	35.82	4.62
MaHsf8	GSMUA_Achr5G15900_001	chr5:12441425..12447734	1644	547	60.12	4.48
MaHsf9	GSMUA_Achr8G27750_001	chr8:30793682..30794765	666	221	25.67	10.22
MaHsf10	GSMUA_Achr3G15640_001	chr3:16716791..16718898	1362	453	51.44	4.66
MaHsf11	GSMUA_Achr6G31750_001	chr6:31438111..31442524	1461	486	55.08	6.52
MaHsf12	GSMUA_Achr9G05660_001	chr9:3615295..3616142	291	96	10.25	4.54
MaHsf13	GSMUA_Achr6G27750_001	chr6:28159817..28165177	1182	393	43.98	4.89
MaHsf14	GSMUA_Achr8G28330_001	chr8:31191645..31193412	984	327	36.82	5.96
MaHsf15	GSMUA_Achr9G01640_001	chr9:1270926..1272784	1146	381	43.15	6.10
MaHsf16	GSMUA_Achr5G03000_001	chr5:2057938..2059891	1005	334	38.81	4.82
MaHsf17	GSMUA_Achr8G20520_001	chr8:25701424..25704747	873	290	33.62	5.03
MaHsf18	GSMUA_Achr1G27200_001	chr1:23837629..23840426	1002	333	38.93	4.95
MaHsf19	GSMUA_Achr6G24430_001	chr6:25125348..25128181	927	308	35.69	5.76
MaHsf20	GSMUA_Achr10G30120_001	chr10:32380864..32382985	912	303	33.25	8.23
MaHsf21	GSMUA_Achr3G06990_001	chr3:4701568..4703325	678	225	25.83	9.63
MaHsf22	GSMUA_Achr7G18900_001	chr7:21734212..21736267	660	219	24.78	8.73
MaHsf23	GSMUA_Achr9G00910_001	chr9:667647..668763	849	282	31.71	6.09

MaHsf24	GSMUA_Achr1G03910_001	chr1:3257650..3259039	873	290	32.83	6.30
MaHsf25	GSMUA_Achr1G07880_001	chr1:6069800..6071502	1227	408	46.02	9.63
MaHsf26	GSMUA_Achr1G16240_001	chr1:12142257..12143417	912	303	33.23	5.51
MaHsf27	GSMUA_Achr4G19760_001	chr4:20610509..20611652	843	280	30.92	7.02
MaHsf28	GSMUA_Achr4G29100_001	chr4:27212139..27213416	816	271	28.95	8.07
MaHsf29	GSMUA_Achr2G11880_001	chr2:14494021..14497735	1563	520	56.91	8.31
MaHsf30	GSMUA_Achr11G10320_001	chr11:8129873..8130879	792	263	30.41	7.79
MaHsf31	GSMUA_Achr4G10060_001	chr4:7368863..7369800	792	263	30.12	6.86
MaHsf32	GSMUA_Achr5G07840_001	chr5:5619472..5620340	783	260	29.90	7.15
MaHsf33	GSMUA_Achr3G07020_001	chr3:4738802..4742102	966	321	35.61	8.61
MaHsf34	GSMUA_Achr9G15370_001	chr9:10071106..10072217	939	312	34.53	8.05
MaHsf35	GSMUA_Achr7G13040_001	chr7:10520835..10522787	1248	415	45.91	9.94
MaHsf36	GSMUA_Achr10G25230_001	chr10:29323648..29324969	759	252	28.56	7.56
MaHsf37	GSMUA_Achr10G27810_001	chr10:30791163..30792852	918	305	33.80	5.48
MaHsf38	GSMUA_Achr4G30990_001	chr4:28352575..28353701	870	289	31.99	5.64
MaHsf39	GSMUA_Achr2G15590_001	chr2:16827154..16827951	663	220	25.25	11.84
MaHsf40	GSMUA_Achr3G15740_001	chr3:16770642..16771558	831	276	31.60	10.46
MaHsf41	GSMUA_Achr8G27820_001	chr8:30822587..30827118	1092	363	40.86	8.70
MaHsf42	GSMUA_Achr3G31860_001	chr3:29984181..29985244	975	324	36.59	6.18
MaHsf43	GSMUA_Achr7G24790_001	chr7:26647113..26648366	630	209	23.90	10.18
